# Conservation of Reactive Stabilization Strategies in the Presence of Step Length Asymmetries During Walking

**DOI:** 10.3389/fnhum.2018.00251

**Published:** 2018-06-27

**Authors:** Chang Liu, Lucas De Macedo, James M. Finley

**Affiliations:** ^1^Department of Biomedical Engineering, University of Southern California, Los Angeles, CA, United States; ^2^Departamento de Engenharia Eletrica, Universidade de Brasília, Brasília, Brazil; ^3^Division of Biokinesiology and Physical Therapy, University of Southern California, Los Angeles, CA, United States; ^4^Neuroscience Graduate Program, University of Southern California, Los Angeles, CA, United States

**Keywords:** stability, asymmetry, locomotion, reactive control, angular momentum

## Abstract

The ability to maintain dynamic balance in response to unexpected perturbations during walking is largely mediated by reactive control strategies. Reactive control during perturbed walking can be characterized by multiple metrics such as measures of whole-body angular momentum (WBAM), which capture the rotational dynamics of the body, and through Floquet analysis which captures the orbital stability of a limit cycle attractor. Recent studies have demonstrated that people with spatiotemporal asymmetries during gait have impaired control of whole-body dynamics as evidenced by higher peak-to-peak ranges of WBAM over the gait cycle. While this may suggest that spatiotemporal asymmetries could impair stability, no studies have quantified how direct modification of asymmetry influences reactive balance control. Here, we used a biofeedback paradigm that allows participants to systematically adopt different levels of step length asymmetry to test the hypothesis that walking asymmetrically impairs the reactive control of balance. In addition, we tested the hypothesis that perturbations to the non-dominant leg would cause less whole-body rotation due to its hypothesized role in weight support during walking. We characterized reactive control strategies in two ways. We first computed integrated angular momentum to characterize changes in whole-body configuration during multi-step responses to perturbations. We also computed the maximum Floquet multipliers (FMs) across the gait cycle, which represent the rate of convergence back to limit cycle behavior. Our results show that integrated angular momentum during the perturbation step and subsequent recovery steps, as well as the magnitude of maximum FMs over the gait cycle, do not change across levels of asymmetry. However, our results showed both limb-dependent and limb-independent responses to unexpected perturbations. Overall, our findings suggest that there is no causal relationship between step length asymmetry and impaired reactive control of balance in the absence of neuromotor impairments. Our approach could be used in future studies to determine if reducing asymmetries in populations with neuromotor impairments, such people post-stroke or amputees improves dynamic stability.

## Introduction

One of the primary challenges for human locomotion is to maintain balance when faced with internally generated or externally imposed perturbations. Two balance control strategies are generally used during locomotion: proactive and reactive control of balance (Patla, 1993). While proactive or feedforward control involves the use of predictions of impending perturbations to avoid falling, reactive control of balance involves the use of feedback about the body’s state to generate balance correcting responses (Patla, 1993; Tang et al., 1998). One of the primary ways in which the reactive control of balance is studied is by applying perturbations during walking and characterizing the resulting perturbation recovery strategies.

Several metrics have been used to quantify balance during locomotion including measures of variability (Stergiou and Decker, 2011), measures derived from nonlinear dynamics such as the maximum Lyapunov exponent (Dingwell and Cusumano, 2000; Dingwell et al., 2001) and long-range correlations (Hausdorff et al., 1996), and biomechanical measures such as dynamic margins of stability (Hof et al., 2005; Hof, 2008). For a detailed review of metrics used to assess dynamic stability during gait, see Bruijn et al. (2013). While each of these methods is useful for characterizing features of control in the presence of instability, we are particularly interested in measures that directly capture whole-body dynamics. One such measure, whole-body angular momentum (WBAM), can be used to capture the body’s response to perturbations and reflects the net contribution of all body segments to the body’s rotation about a given axis. WBAM is highly regulated during normal human locomotion (Popovic et al., 2004; Herr and Popovic, 2008) as the peak-to-peak range of WBAM about the body’s center of mass is much smaller than the angular momentum of single segments due to momentum cancellation between the limbs (Herr and Popovic, 2008). In a recent study, Martelli et al. (2013) used WBAM to characterize recovery strategies in response to multidirectional perturbations during walking in healthy individuals. They found that perturbations resulted in increased angular momentum and subsequent compensatory reactions.

Another metric used to characterize dynamic stability is the maximum Floquet multiplier (FM) which is commonly used to assess the rate of divergence/convergence from a fixed point, characterized by a kinematic state vector, in response to small perturbations (Hurmuzlu and Basdogan, 1994; Kuo, 1999; Dingwell and Kang, 2007). This measure is based on the fact that human walking is strongly periodic and can be characterized as a limit cycle attractor. Previous studies have established that the maximum FM remains below one during unperturbed walking (Hurmuzlu and Basdogan, 1994; Dingwell and Kang, 2007; Granata and Lockhart, 2008; Bruijn et al., 2009) which indicates that small perturbations always converge toward a limit cycle. The maximum FM increases when walking in destabilizing environments, but still remains below one as people are able to use proactive and reactive control to maintain balance (McAndrew et al., 2012). Both WBAM and the maximum FM capture a different aspect of reactive control during walking and together provide a detailed description of the control of dynamic balance.

The ability to successfully restore balance is vital for populations with gait asymmetries such as people post-stroke (Chen et al., 2005; Balasubramanian et al., 2007; Allen et al., 2011), unilateral amputees (Barth et al., 1992; Underwood et al., 2004; Zmitrewicz et al., 2006) and patients with ACL reconstruction (Winiarski and Czamara, 2012). However, these populations are known to have balance deficits during walking. For example, Lewek et al. (2014) examined the relationship between spatiotemporal gait asymmetry and balance in people post-stroke and showed that step length asymmetries were correlated with scores on the Berg Balance Scale, suggesting that gait asymmetries are associated with fall risk in these individuals. In addition, recent studies have demonstrated that people post-stroke have impaired control of whole-body dynamics as captured by higher peak-to-peak ranges of WBAM (Nott et al., 2014; Vistamehr et al., 2016) and reductions in local and orbital stability (Kao et al., 2014). Likewise, unilateral amputees demonstrate a greater range of angular momentum during the half of the gait cycle from foot contact of the residual limb to contact of the intact limb and a smaller range of angular momentum during the second half of the gait cycle due to reduced leg propulsion in the sagittal plane (Silverman and Neptune, 2011). Although these studies have demonstrated an association between asymmetry and measures of stability, it remains to be seen if spatiotemporal asymmetry alone is causally associated with stability in the absence of neuromotor impairments.

In addition to an effect of asymmetry, the reactive control of stability may also be impacted by limb dominance. There is evidence suggesting that the dominant leg generates more propulsion during walking while the non-dominant leg preferentially provides support (Sadeghi et al., 1997). Õunpuu and Winter (1989) found that the normalized EMG amplitude of most plantar flexor muscles was greater in the dominant limb, which may reflect its preferential role in propulsion generation. Also, Martelli et al. (2013) demonstrated that recovery of WBAM about the roll-axis in response to a perturbation depends on the side of the perturbation. Specifically, the reactive responses to perturbations on the non-dominant side, as captured by the principal components of the segmental angular momenta, were more similar to pre-perturbation behavior than responses following perturbations to the dominant side. Thus, the nondominant leg may be better suited for maintaining stability in response to perturbations.

The objectives of this study are to quantify how direct modification of spatiotemporal asymmetry influences the reactive control of balance during walking and to determine whether the reactive control of balance is influenced by limb dominance. We hypothesized that: (1) modifications of step length asymmetry will impair the control of whole-body rotation and increase the maximum FM during unexpected perturbations which, together, would indicate that the reactive control of balance is compromised by asymmetry; and (2) that perturbations of the non-dominant leg would produce less whole-body rotation due to this limb’s proposed role in providing stability during locomotion (Sadeghi et al., 1997). Here, we chose to use the maximum FM to quantify orbital stability because it allowed us to capture differences in stability throughout the gait cycle. These hypotheses were tested by using visual feedback to induce changes in step length asymmetry during walking and imposing slip-like perturbations on a dual-belt treadmill. Our findings may inform our understanding of how interventions aimed at improving symmetry in populations with neuromotor impairments may impact balance control.

## Materials and Methods

### Participant Characteristics

A total of 19 healthy young individuals (10 M, 24 ± 4 years old) with no musculoskeletal or gait impairments participated in this study. Lower limb dominance was determined by asking participants which leg they would use to kick a ball. This study was carried out in accordance with the recommendations of the Declaration of Helsinki with written informed consent from all subjects. The protocol was approved by the Institutional Review Board of the University of Southern California.

### Experiment Protocol

**Figure 1:**
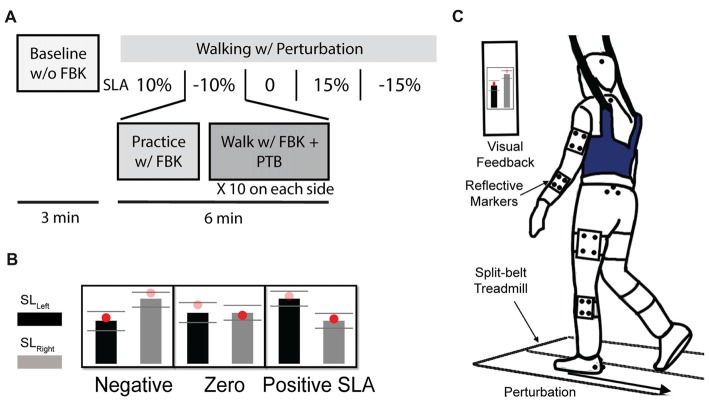
**(A)** Experiment protocol. Participants completed a total of six trials. Participant’s Baseline step length asymmetry was collected during the first 3-min baseline trial without visual feedback. Then, they were instructed to complete a randomized sequence of five 6-min trials with target step length asymmetries of 0%, ±10% and ±15%. During each visual feedback trial, the participant first practiced with feedback for 1 min, then 10 perturbations were randomly applied at foot strike on each side. **(B)** Visual feedback for three of the five trials of step length asymmetry are shown. **(C)** Experimental setup. Participant were instructed to walk on the split-belt treadmill. A “success” message would appear on the screen when step length was within the three standard deviations of the desired target.

The purpose of this study was to assess whether changes in step length asymmetry affect the reactive control of balance during walking. Participants completed six separate trials walking on an instrumented, dual-belt treadmill at 1.0 m/s (Bertec, Columbus, OH, USA) and reacted to unexpected accelerations of the treadmill belts throughout the experiment (Figure 1A). For the first trial, participants walked on the treadmill for 3-min (Baseline) to obtain their natural level of step length asymmetry. Then, for subsequent trials, visual feedback indicating the desired step lengths was provided to assist participants in actively modifying their asymmetry relative to their natural step length asymmetry. Participants completed a randomized sequence of five 6-min trials with target step length asymmetries (SLA, Eq. 1) of 0%, ±10% and ±15% where 0% represents each participant’s baseline SLA.

(1) $$SLA=100*\frac{{\text{SL}}_{\text{left}}-{\text{SL}}_{\text{right}}}{{\text{SL}}_{\text{left}}+{\text{SL}}_{\text{right}}}$$

Participants viewed the step length targets on a computer monitor attached to the treadmill post (Figure 1B). During each trial, participants first practiced walking at a given SLA with visual feedback for 1 min before experiencing any perturbations (Figure 1C). A “success” message would appear on the screen when the achieved step length was within the three standard deviations of the desired target length. The standard deviation for each target was determined on an individual basis from each participant’s Baseline step length variability. Participants were encouraged to maintain the desired SLA and get as many success messages as possible.

**Figure 2:**
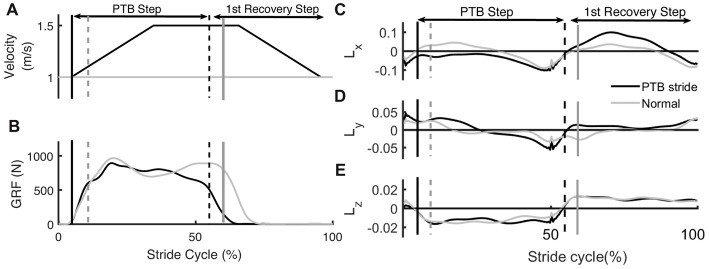
Example of time series data from an unperturbed and perturbed step. **(A)** Treadmill belt velocity, **(B)** vertical ground reaction force and **(C–E)** whole-body angular momentum (WBAM) for a representative perturbation step and recovery stride. The gray traces indicate the time series data for an unperturbed stride while the black traces indicate a perturbation stride. Each stride begins at heel strike. Black vertical lines correspond to the time of foot strike and gray vertical lines correspond to time of toe-off. Solid lines and dashed lines represent contralateral legs.

Step length was estimated during the experiment as the anterior/posterior distance between the center of pressure on the left and right force plates at foot strike. Foot strike was defined as the point when the vertical ground reaction force became greater than 150 N. For the trials with visual feedback, 10 unexpected perturbations, where the treadmill accelerated to 1.5 m/s, were randomly applied to each side (right or left). Each perturbation was remotely triggered by preprogrammed Python code such that the participants could not anticipate when the perturbation would occur. During pilot testing, we found that there was approximately a 200 ms time delay between when the perturbation signal was sent to the treadmill and when the treadmill began accelerating. Thus, during the first minute of practice for each trial, we calculated each individual’s average right and left step times. We then triggered the perturbations 200 ms before the predicted foot strike of the perturbed leg so that the acceleration of treadmill would coincide with foot strike. All perturbations were characterized by a trapezoidal speed profile in which the speed increased at foot strike to 1.5 m/s at an acceleration of 1.6 m/s^2^, was held for 0.3 s, and then decelerated back to 1.0 m/s at 1.6 m/s^2^ during swing phase of the perturbed leg (Figure 2A). These parameters were selected based on results from a series of pilot tests which demonstrated that these perturbations were sufficient to elicit both changes in step length asymmetry and changes in WBAM. The belt speed was held for 0.3 s to ensure that the belt speed did not decelerate before the toe-off of the perturbed leg. The perturbations randomly occurred within a range of 20–30 steps after the previous perturbation to provide participants with enough time to reestablish their normal walking pattern.

### Data Acquisition

A 10-camera Qualysis motion capture system (Qualysis AB, Gothenburg, Sweden) recorded 3D marker kinematics at 100 Hz and ground reaction forces (Figure 2B) at 1000 Hz. A set of 19 mm spherical markers were placed on anatomical landmarks to create a 13-segment, full-body model (Song et al., 2012; Havens et al., 2018). Marker clusters were placed on the upper arms, forearms, thighs, shanks, and the back of heels. At the beginning of each trial, marker positions were calibrated during a 5-s standing trial. All joint markers were removed after the calibration.

### Data Processing

Kinematic and kinetic data were post-processed in Visual3D (C-Motion, Rockville, MD, USA) and Matlab 2017a (Mathworks, Natick, MA, USA) to compute variables of interest. Marker position data and ground reaction forces were low-pass filtered by 4th order Butterworth filters with cutoff frequencies of 6 Hz and 20 Hz respectively. The type of filter and cutoff frequency were selected based on previous literature (Reisman et al., 2009; Winter, 2009; Kurz et al., 2012). The timing of each perturbation relative to foot strike was reexamined in Matlab. If the treadmill belt did not accelerate during the 300 ms window around foot strike (from 150 ms before to 150 ms after foot strike), the perturbation was excluded from analysis. On average, approximately 4 of 20 perturbations were excluded for each trial.

In order to account for differences between the target and achieved SLA, we calculated achieved SLA as follows: first, we calculated the mean SLA of four strides before each perturbation and then distributed them into five equally spaced bins centered at −15%, −10%, 0, 10%, 15% with a bin width equal to 5%. We used this re-categorized SLA as the independent variable in our statistical analyses instead of target SLA.

#### Whole-Body Angular Momentum

WBAM was computed to determine how the rotational behavior of the body changed in response to the treadmill perturbations. In order to calculate WBAM, a 13-segment full-body model was first created using Visual 3D (C-Motion, Rockville, MD, USA). The segments of the model included the head, trunk, pelvis, upper arms, forearms, thighs, shanks and feet. Each limb segment’s mass was modeled based on anthropometric tables (Dempster, 1955). Segment geometry was modeled based on the description in (Hanavan, 1964). The trunk and pelvis were modeled as elliptical cylinders, the head was modeled as an ellipsoid, and all the other segments were modeled as circular cones. All segments had six degrees of freedom, and no connecting joints (i.e., constraints between segments) were defined. Segmental linear and angular velocity were computed in Visual 3D using the filtered marker position data. WBAM (*L*) was then computed as the sum of all segmental angular momenta which were composed of segmental rotation about the body’s center of mass and rotation of each segment about its own center of mass (Silverman and Neptune, 2011). Then, *L* was normalized by the participant’s mass (*M*), treadmill velocity (*V*), and the participant’s height (*H*) (Eq. 2).

(2) $$\vec{L}=\frac{\sum_{i}\left[m_{i}\left({\vec{r}}_{CM-i}^{i}\times {\vec{v}}_{CM-i}^{i}\right)+I^{i}\omega^{i}\right]}{MVH}$$

Here, *m* is segmental mass, *r* is the distance from segment to the body COM, *I* is the segmental moment of inertia, ω is segmental angular velocity, and the index *i* corresponds to individual limb segments. The coordinate system for analysis of angular momentum was defined as follows: the *x*-axis was the pitch axis and positive to the right, the *y*-axis was the roll axis and positive in the anterior direction, and the *z*-axis was the yaw axis and positive in the vertical direction. WBAM for each stride cycle (from the foot strike on one side to the subsequent foot strike on the same side) was normalized to 100 points. In addition, integrated WBAM (*L*_int_) was computed as the area under the curve of the WBAM trajectory for each step cycle to quantify the degree to which the body rotates about its center of mass across a step cycle.

#### Orbital Stability

We used Floquet analysis (Hurmuzlu and Basdogan, 1994; Hurmuzlu et al., 1996; Kuo, 1999; Dingwell and Kang, 2007; Hobbelen and Wisse, 2007; Kurz et al., 2012; Bruijn et al., 2013) to determine how orbital stability was affected by walking with different levels of SLA. Two participants were excluded from this analysis as there was a break in one of their trials and therefore, we did not have continuous data for the analysis. For this analysis, we used WBAM data as computed in the “Whole-Body Angular Momentum” section. First, state vectors (*S*) at each time point in the normalized gait cycle were constructed from the WBAM signal and its first derivative (Eq. 3). Then, Poincare maps (Eq. 4) were defined at each section of the gait cycle.

(3) $$S=\;{\left[L_{x}\;L_{y}\;L_{z}\;{\dot{L}}_{x}\;{\dot{L}}_{y}\;{\dot{L}}_{z}\right]}^{T}$$

(4) $$S_{k+1}=F\left(S_{k}\right)$$

Here, *k* is the stride number and *S*_*k*_ are the state vectors of the system.

**Figure 3:**
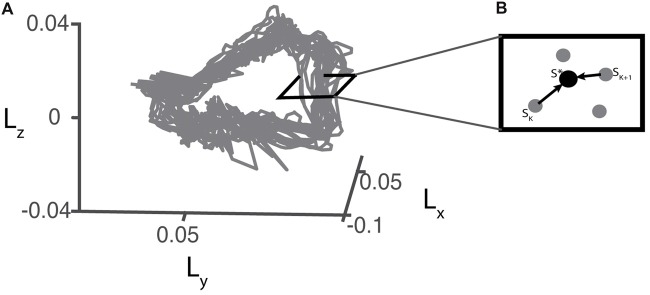
**(A)** Example of a 3D projection of the angular momentum trajectory recorded during baseline walking for one representative participant. **(B)** Illustration of a hypothetical perpendicular slice of the angular momentum trajectory as a Poincare section. S* represents the fixed point which is the average of pre-perturbation strides.

For each trial, we defined the fixed points (S*) (Eq. 5) at each Poincare section by averaging all sets of angular momentum trajectory during the four strides before each perturbation occurred (Figure 3). Stride-to-stride fluctuations about the fixed point allowed us to examine the persistence of deviations from the mean trajectory.

(5) $$S^{*}=F\left(S^{*}\right)$$

Orbital stability at each Poincare section was assessed by linearly approximating the effects of perturbations that caused deviations from the fixed point (Eq. 6). For all trials, we used 250 strides to compute FMs based on previous literature which established that at least 150 strides were necessary to precisely measure FM (Bruijn et al., 2009).

(6) $$\left[S_{k+1}-S^{*}\right]\;\approx J\left(S^{*}\right)\left[S_{k}-S^{*}\right]$$

Here, *J* is the Jacobian matrix estimated using the pseudo inverse at each Poincare section (Kurz et al., 2012). *S_k_* − S* represents deviations from the fixed point. FM were calculated as the eigenvalues of Jacobian matrix (*J*(S*)), and we selected the maximum value of the FM (FM_max_) to assess orbital stability (Dingwell and Kang, 2007). If the magnitude of FM_max_ < 1, the system is orbitally stable, otherwise, the system is unstable (Hurmuzlu and Basdogan, 1994; Dingwell and Kang, 2007; Bruijn et al., 2013). We computed FM_max_ for each Poincare section (each % gait cycle) to determine how stability changes over a stride cycle. We also determined FM_max_ across the entire gait cycle for further statistical analysis as this value represents the most unstable point during the gait cycle (Dingwell and Kang, 2007).

### Statistical Analysis

All statistical analyses were performed in Matlab R2017a (Mathworks, Natick, MA, USA). Repeated measures analysis of variance (RM-ANOVA) was used to determine if values of integrated angular momentum about each axis for the steps after the perturbation differed from values during baseline steps. *Post hoc* comparisons used the Tukey-Kramer correction for multiple comparisons.

Linear mixed-effect models were fit to examine the relationship between independent variables achieved SLA (Asym) and side of perturbation (Side) and dependent variables *L*_int_ about each axis to determine how the effect of perturbations varied with asymmetry and between limbs. This model included main effects for Asym and Side as well as an interaction between Asym and Side to determine whether the effect of asymmetry depended on the side of the perturbation. The linear mixed-effect models were fit for four consecutive steps (Baseline, Perturbation, Recovery 1 and Recovery 2) for each axis. The integrated angular momentum for the trial with an SLA of zero was selected as the reference level. We used a mixed effect model instead of a RM-ANOVA for this analysis because the number of observations at each level of achieved asymmetry was unequal.

Similarly, a linear mixed effect model was fit to represent the relationship between target asymmetry (independent variable) and the FM_max_ (dependent variable) in order to see if the orbital stability was associated with target asymmetry. For both sets of analyses, models including random intercepts and/or slopes were compared against a model with only fixed effects and the most parsimonious model was chosen based on the results of a likelihood ratio test.

## Results

**Figure 4:**
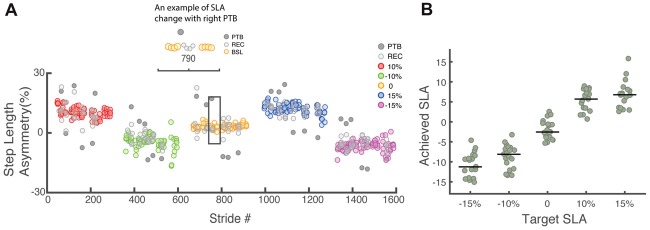
**(A)** Raw step length asymmetry data for one representative participant. Each data point represents the step length asymmetry. The target asymmetries for this example followed the order of 10%, −10%, 0, 15%, −15%. Each target asymmetry is represented by a different color. BSL: baseline step; PTB: perturbation step; REC: recovery step. **(B)** Achieved step length asymmetry vs. target step length asymmetry for all participants (*N* = 19). Achieved step length asymmetry is calculated as the average of all pre-perturbation strides and tends to undershoot the target at 15% and −15%. The green dots represent individual data. Horizontal bars indicate the median across all participants.

Participants were able to update and maintain their SLA for the full duration of each trial (~6 min) using visual feedback and recover from deviations in SLA resulting from the perturbations (Figure 4A). If the perturbation occurred on the right leg, the left leg would step further forward to recover from the perturbation and SLA would increase based on Equation 1. On the other hand, if the perturbation was occurred on the left leg, SLA for the perturbation stride became more negative. The achieved SLA (Figure 4B) was calculated relative to baseline asymmetry of 1.7 ± 3%. There was considerable variability in the achieved asymmetry across participants, especially when the target SLA was large. The average residual between achieved SLA and target SLA (|SLA_target_ − SLA_achieved_|) across all participants was 4.7 ± 2.9%, 2.9 ± 1.7%, 2.4 ± 1.6%, 4.3 ± 2.8%, 7.7 ± 3.5% for −15%, −10%, 0, 10%, 15% target SLA respectively. Our analysis used participants’ achieved asymmetry rather than target asymmetry to better reflect their actual performance. The total number of perturbations in each step length asymmetry bin were as follows: 110 perturbations for −15%, 314 perturbations for −10%, 265 perturbations for −5%, 294 perturbations for 0%, 285 perturbations for 5%, 191 perturbations for 10% and 69 perturbations for 15% SLA.

### Modulation of Whole-Body Angular Momentum in Response to Treadmill Perturbations

Measures of WBAM varied systematically across trials. We measured the WBAM about three axes to better understand how participants react to the perturbations. The rapid acceleration of the belts caused consistent, immediate effects on WBAM and triggered multi-step balance recovery responses. The immediate effect was most obvious along the direction of perturbation (pitch axis, Figure 2C). During the perturbation step, angular momentum became more negative as the body rotated forward (−pitch). In order to compensate for the perturbation, participants increased the length of the subsequent step to generate positive angular momentum and initiate backward rotation (+pitch). Deviations in body rotation about the roll and yaw axes relative to unperturbed walking were less prominent (Figures 2D,E).

**Figure 5:**
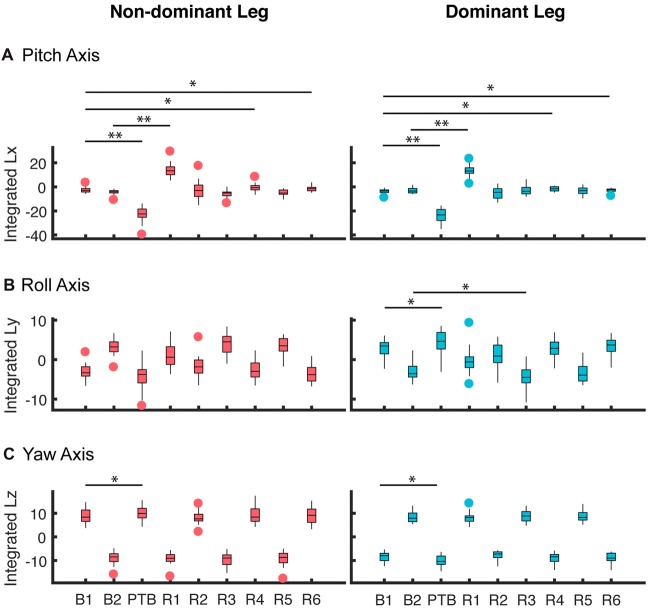
Averaged integrated angular momentum over the step cycle for all participants about the **(A)** pitch, **(B)** roll and **(C)** yaw axes for perturbations that occurred on the non-dominant (left column) and dominant side (right column). These results represent the 0% asymmetry condition (*N* = 19). The first step (B1) corresponds to the non-dominant limb for the left column and the dominant limb for the right column. Subsequent steps alternate between non-dominant and dominant. B: Baseline; PTB: Perturbation; R: Recovery. The horizontal bars and corresponding stars indicated whether the difference in integrated angular momentum between two steps was significant (**p < 0.001, *p < 0.05). The data are represented as boxplots such that the lower and upper edges of the box indicate the 25th and 75th percentile of the data, respectively. The horizontal line within each box indicates the median. The whiskers extend to the furthest data point beyond the lower or upper edges of the box that is within a distance of 1.5 times the middle 50th percentile of the data. Points that lie beyond the whiskers denote outliers.

To quantify the effects of the perturbations on whole-body configuration, we computed the integrated angular momentum across the step cycle (Figure 5). When walking symmetrically, the integrated angular momentum was relatively small during baseline walking and showed little step-to-step variability about the pitch axis. For the roll axis, positive and negative values of *L*_int_ correspond to transitions from the right to left leg and from the left to right leg, respectively. For the yaw axis, positive and negative values of *L*_int_ correspond to transitions from the left to the right leg and from the right to left leg, respectively. During the perturbation step, there was a significant increase in integrated angular momentum about the pitch axis which reflected the increase in the body’s forward rotation. A repeated measures ANOVA was used to determine when the participants recovered from the perturbation when walking symmetrically. We found a main effect of step number on integrated angular momentum for the pitch axis (RM-ANOVA, *F* = 185.5, *p* < 0.001), and a significant interaction between step number and perturbation side for roll axis (*F* = 58.7, *p* < 0.001), and yaw axis (*F* = 434.76, *p* < 0.001). For the pitch axis, *post hoc* analysis revealed that *L*_int_ differed from baseline during the perturbation (PTB) step (*p* < 0.001 both sides), recovery (R) steps R1 (*p* < 0.001 both sides), R4 (*p* = 0.006 Dominant side, *p* = 0.002 Non-dominant side) and R6 (*p* = 0.013 Dominant side, *p* = 0.001 Non-dominant side). About the roll axis, significant differences in *L*_int_ were found during the PTB step (*p* = 0.001) and R3 (*p* = 0.001), but only on the dominant side. Lastly, about the yaw axis, a significant difference was found at perturbation step (*p* < 0.001) for both sides.

### Effects of Asymmetry on the Reactive Control of Whole-Body Angular Momentum

**Figure 6:**
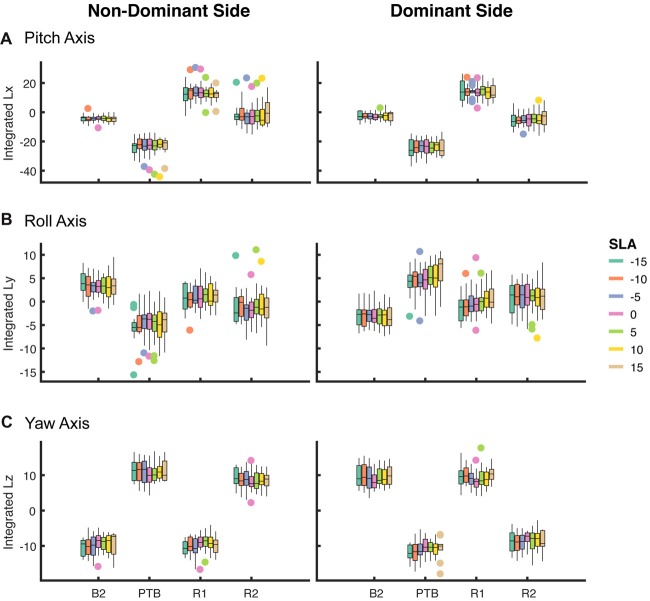
Box plot of integrated angular momentum about the **(A)** pitch, **(B)** roll and **(C)** yaw axes at baseline step (B2), perturbation step (PTB) and recovery steps (R1 and R2) across each level of achieved asymmetry (*N* = 19) for perturbations on the non-dominant (left column) and dominant (right column) sides.

Next, we asked whether walking with asymmetric step lengths would have negative effects on participant’s reactive control of balance (Figure 6). We fit a linear mixed effect model relating asymmetry to the integrated angular momentum at each step (Final Baseline step (B2), PTB, R1 and R2) and selected the simplest model based on a likelihood ratio test. An SLA of zero was selected as the reference level. The model indicated that a random intercept was necessary to account for individual differences between participants (*p* < 0.001). There were no significant main effects found for Asymmetry, Side (pitch axis) or the interaction between Asymmetry and Side during baseline (B2) steps (Table 1). Similarly, we examined whether asymmetry influenced measures of integrated angular momentum at the PTB step and found no significant main effects for Asymmetry, Side or the interaction between Asymmetry and Side during any of these steps (Table 1). Lastly, we examined whether asymmetry influenced integrated angular momentum during the first or second recovery steps (R1 and R2). There were no significant main effects found for Asymmetry, Side or the interaction between Asymmetry and Side during first or second recovery steps (Table 1). The significance found for Side in roll and yaw axis was due to differences in the direction of body rotation at each step. Overall, these results indicate that imposed asymmetry does not have a systematic effect on the reactive control of balance as assessed by measures of WBAM.

**Table 1:** Statistical results from the ANOVA examining the effects of asymmetry and perturbation side on integrated whole-body angular momentum (WBAM) for each step type.

Step type	Axis	Factor	DF1	DF2	*F* value	*p* value
*B2*	Pitch	Asymmetry	6	210	0.10	0.99
		Side	1	210	2.84	0.09
		Asymmetry:Side	6	210	0.18	0.98
	Roll	Asymmetry	6	210	0.31	0.93
		Side	1	210	76.10	<0.001
		Asymmetry:Side	6	210	0.24	0.96
	Yaw	Asymmetry	6	210	0.72	0.63
		Side	1	210	397.00	<0.001
		Asymmetry:Side	6	210	0.96	0.45
*PTB*	Pitch	Asymmetry	6	211	0.25	0.96
		Side	1	211	0.30	0.59
		Asymmetry:Side	6	211	0.59	0.77
	Roll	Asymmetry	6	211	0.39	0.89
		Side	1	211	85.00	<0.001
		Asymmetry:Side	6	211	0.41	0.87
	Yaw	Asymmetry	6	211	0.38	0.89
		Side	1	211	577.00	<0.001
		Asymmetry:Side	6	211	1.33	0.25
*R1*	Pitch	Asymmetry	6	211	0.51	0.80
		Side	1	211	0.31	0.60
		Asymmetry:Side	6	211	0.76	0.60
	Roll	Asymmetry	6	211	0.36	0.90
		Side	1	211	2.85	0.09
		Asymmetry:Side	6	211	0.39	0.89
	Yaw	Asymmetry	6	211	1.00	0.42
		Side	1	211	491.00	<0.001
		Asymmetry:Side	6	211	1.57	0.16
*R2*	Pitch	Asymmetry	6	211	0.90	0.5
		Side	1	211	2.70	0.1
		Asymmetry:Side	6	211	0.21	0.97
	Roll	Asymmetry	6	211	0.65	0.69
		Side	1	211	6.39	0.01
		Asymmetry:Side	6	211	0.00	0.98
	Yaw	Asymmetry	6	211	0.47	0.83
		Side	1	211	386.00	<0.001
		Asymmetry:Side	6	211	0.92	0.48

*B2, Baseline step; PTB, Perturbation Step; R1, First Recovery Step; R2, Second Recovery Step*.

### Orbital Stability

**Figure 7:**
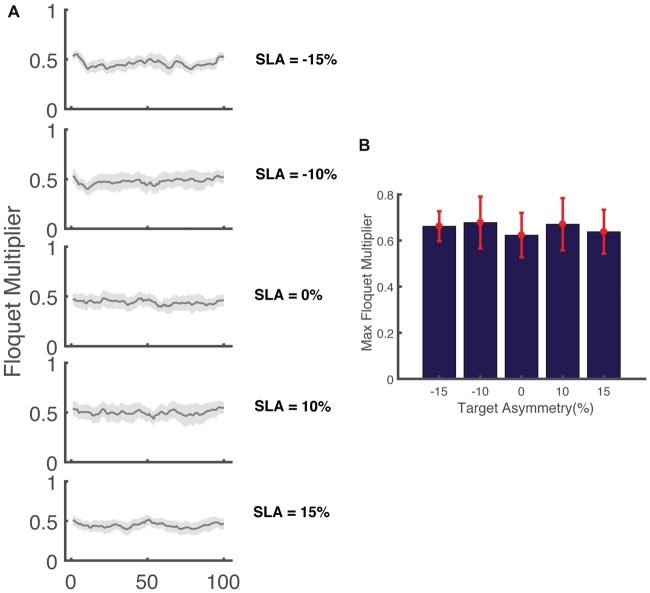
**(A)** Variation in the magnitude of the maximum Floquet multiplier (FM) across the gait cycle for five levels of target asymmetry (*N* = 17). The shaded area indicates the 95% confidence interval. **(B)** FM_Max_ across all levels of asymmetry for (*N* = 17) participants.

To further investigate how asymmetry impacts dynamic stability, we performed Floquet analysis to determine if asymmetry influenced measures of orbital stability during perturbed walking. Our results show that the FM_Max_ from all five trials was less than one indicating that participants remained orbitally stable in spite of the perturbations that occurred while walking (Figure 7). The range of the FM computed across the stride cycle at 0% SLA was 0.41 ± 0.09 across all participants, which is similar, but slightly smaller than that reported by Dingwell and Kang (2007) (~0.5) when participants walked on the treadmill in the absence of perturbations. Similar to our results for integrated angular momentum, there was no association between the SLA and measures of orbital stability (*F*_(4,80)_ = 0.86, *p* = 0.5). The FM_Max_ across all asymmetries were 0.61 ± 0.09, 0.63 ± 0.13, 0.57 ± 0.11, 0.62 ± 0.13 and 0.59 ± 0.09 for target asymmetries of −15%, −10%, 0, 10% and 15%, respectively.

## Discussion

### Conservation of Reactive Response

This study asked the question of how step length asymmetry affects the reactive control of balance during walking. Previous studies have demonstrated that people with gait asymmetries have impairments in dynamic balance leading to the possibility that asymmetry itself is sub-optimal for balance control. Here, we hypothesized that asymmetry would impair the reactive control of balance, and we tested this hypothesis by imposing different levels of step length asymmetry and characterizing participants’ response to perturbations. Although we consistently elicited reactive responses to regain balance, we rejected our primary hypothesis that asymmetry impairs the reactive control of balance as no significant difference in WBAM was found across levels of asymmetry. In addition, Floquet analysis revealed that orbital stability was well maintained and did not vary systematically with different levels of asymmetry. These results indicate that reactive control of stability may be well controlled by healthy people even when they change their preferred walking pattern to walk asymmetrically.

A potential explanation for the discrepancy between our hypothesis and the observed results is that participants may have chosen a more conservative strategy due to the novel study demands. In other words, participants may have taken more cautious, wider steps to increase their base of support (Woollacott and Tang, 1997) when asked to walk asymmetrically and subsequently improved the proactive control of stability during the task. However, we found no difference in step width between levels of target asymmetry. Thus, there does not seem to be strong evidence that participants’ recovery strategies were biased by use of a more conservative pattern of movement.

Another possible reason why we did not observe an effect of asymmetry on the reactive control of stability is that reactive responses to unexpected perturbations may be mediated by neural pathways that generate stereotypical reactive responses that remain invariant across tasks. Previous work by Aprigliano et al. (2017) used principal component analysis (PCA) to analyze coordination between the shank, foot and thigh in response to slip-like perturbations and found that there was no difference in coordination between fall-prone elderly people and healthy young adults. Similarly, Martelli et al. (2013) used PCA of segmental angular momentum to show that the intersegmental coordination patterns observed during compensatory steps are highly correlated with the patterns observed during unperturbed walking. This conservation of whole-body momentum across asymmetries suggests that reactive responses may result from pre-programmed, stereotypical actions that are sufficient to restore the stability (Martelli et al., 2013).

### Effects of Limb-Dominance on Reactive Control of Balance

We also hypothesized that perturbations of the non-dominant leg would produce less whole-body rotation due to the non-dominant limb’s role in maintaining balance. Previous work has shown that the non-dominant leg may preferentially be used to support body weight while the dominant leg may generate more propulsion (Sadeghi et al., 1997). Consistent with this idea, WBAM about the roll axis did not differ across strides for the non-dominant leg while, in contrast, we observed significant differences in *L*_int_ about the roll axis between unperturbed, perturbed, and recovery strides for the dominant leg. This suggests that the non-dominant leg may be better at maintaining medial-lateral balance. However, we also found that there was no significant effect of the side of the perturbation on our measures of integrated angular momentum. This presence of both limb-specific and limb-independent recovery responses requires further investigation to establish the effect of limb dominance on balance recovery.

### Orbital Stability During Asymmetric Walking

In general, populations with a high incidence of falls are shown to have increased orbital instability relative to unimpaired controls as characterized by a larger maximum FM (Hurmuzlu et al., 1996; Granata and Lockhart, 2008; Kurz et al., 2012). In our study, we were interested in determining whether walking with spatiotemporal asymmetry would modulate orbital stability. As the results demonstrated, all participants in our study had orbitally stable walking patterns, regardless of the level of SLA. Also, no difference in FM_Max_ was found during asymmetrical walking compared with symmetric walking. This is in contrast to previous work which shown that voluntarily changing step length reduces orbital stability of human walking (McAndrew Young and Dingwell, 2012). One possible explanation for the discrepancy between the current study and previous work is that our study provided visual feedback for regulating SLA, which may decrease the step length variability compared to (McAndrew Young and Dingwell, 2012). In addition, we chose to maintain a consistent stride length while varying asymmetry whereas the McAndrew Young and Dingwell’s (2012) study involved significant increases in stride length. As a result, Floquet analysis may not be sensitive enough to detect the destabilizing effects of SLA in the absence of changes in stride length. Our findings also contrast previous studies which showed that walking on a pseudo-randomly oscillating treadmill reduced orbital stability (McAndrew et al., 2010; Beurskens et al., 2014). A potential reason for this difference is that our study perturbed the participants with discrete mechanical perturbations at foot contact whereas previous work used continuous perturbations throughout the gait cycle. It is possible that the effect of these discrete perturbations dissipates quickly for healthy participants resulting in negligible changes in orbital stability due to imposed SLA.

Although this study showed that the whole-body reactive response was not affected by the presence of step length asymmetry in healthy participants, it is possible that this result was influenced by the fact that participants were instructed to walk on the treadmill with a fixed speed, which might alter the strategies participants use to generate the desired asymmetries. While Nagano et al. (2011) showed that there were no differences in step lengths when young adults walked on a treadmill or over ground at a given speed, temporal parameters such as double stance time and swing time did differ. In addition, people produce reduced dorsiflexor moments, reduced knee extensor moments, and greater hip extensor moments in the sagittal plane during treadmill walking (Lee and Hidler, 2008), which could also affect the strategies used to modify symmetry on the treadmill. As a result, it would be interesting for future studies to compare the effects of asymmetry on reactive control balance during over ground vs. treadmill walking.

Aside from reactive responses, proactive control also plays a part in maintaining balance on the treadmill in the presence of slip perturbations. Although participants voluntarily changed their SLA to match the visual feedback, modification of SLA did not impair their whole-body balance during unperturbed steps (Figure 6). This might reflect the fact that healthy individuals used novel proactive strategies to maintain a consistent range of WBAM in the presence of step length asymmetry. Another possibility is that after the initial exposure to the treadmill perturbations, participants may have adopted strategies to improve stability and to prepare themselves for the perturbations. Previous work has shown that the central nervous system can adjust its control strategy based on the prior experience to produce a more cautious gait and reduce the risk of balance loss by altering muscle activation and the resulting interaction between the foot and the surface (Heiden et al., 2006). In addition, previous studies have showed that people were able to reduce backward balance loss with exposure to multiple slip perturbations using proactive adjustments during sit-to-stance task and over ground walking (Pai et al., 2003; Bhatt et al., 2006). Analysis of interlimb coordination during baseline trials could be useful to reveal how participants were able to adopt an invariant control of WBAM despite the presence of marked step length asymmetries.

Lastly, although we found that symmetric walking did not necessarily bring benefits for reactive control of balance for healthy subjects, this does not necessarily mean it holds true for fall-prone populations with sensory or motor impairments. These impairments may significantly affect one’s ability to recover from unexpected perturbation. Fall-prone populations such as people post-stroke may suffer from sensorimotor deficits, which prevent them from appropriately sensing perturbations and planning and executing effective responses to regain balance. Although a number of recent studies have shown that it is possible to improve spatiotemporal symmetry in people post-stroke (Reisman et al., 2009; Awad et al., 2016), it remains to be seen if reductions in symmetry improve dynamic balance. The approaches used in this study may help separate the effects of asymmetry on balance from the effects of neuromotor deficits and lead to better informed locomotor training for people post-stroke.

### Limitations

This study had a few limitations. First, there was inconsistency in performance such that the achieved step length tended to undershoot the target at the larger SLA. This is likely because larger asymmetries are energetically costly and may also reflect biomechanical constraints that prevent substantial extension of the hip beyond that observed during normal walking (Sánchez et al., 2017). Another factor that could have affected our analysis of reactive control is that participants were protected by a harness, which may have restricted forward trunk rotation. However, since the harness was slack during the full experiment, we think this is unlikely. Lastly, calculation of FMs requires a linear approximation to compute the effects of perturbations from stride to stride, but the large perturbations in our study may introduce some non-linearity. We think this effect is likely to be negligible as the FMs we computed were consistent with previous work that assessed stability during walking in destabilizing environments (McAndrew et al., 2012).

## Author Contributions

CL designed the experiment, collected data, analyzed data and wrote the manuscript. LM designed the experiment, collected preliminary data and helped develop the procedure for data processing and analysis. JF conceived of the experiment, advised in data analyses and edited the manuscript.

## Conflict of Interest Statement

The authors declare that the research was conducted in the absence of any commercial or financial relationships that could be construed as a potential conflict of interest.
